# A systematic literature review on the role of glial cells in the pathomechanisms of migraine

**DOI:** 10.3389/fnmol.2023.1219574

**Published:** 2023-06-30

**Authors:** Shanshan Zhang, Justin Azubuine, Christian Schmeer

**Affiliations:** Department of Neurology, Jena University Hospital, Jena, Germany

**Keywords:** migraine, astrocyte, microglia, satellite glial cell, Schwann cell, Calcitonin Gene-Related Peptide (CGRP)

## Abstract

**Background:**

The pathomechanisms underlying migraine are intricate and remain largely unclear. Initially regarded as a neuronal disorder, migraine research primarily concentrated on understanding the pathophysiological changes within neurons. However, recent advances have revealed the significant involvement of neuroinflammation and the neuro-glio-vascular interplay in migraine pathogenesis.

**Methods:**

A systematic search was conducted in PubMed, Scopus, and Web of Science databases from their inception until November 2022. The retrieved results underwent a screening process based on title and abstract, and the full texts of the remaining papers were thoroughly assessed for eligibility. Only studies that met the predetermined inclusion criteria were included in the review.

**Results:**

Fifty-nine studies, consisting of 6 human studies and 53 animal studies, met the inclusion criteria. Among the 6 human studies, 2 focused on genetic analyses, while the remaining studies employed functional imaging, serum analyses and clinical trials. Regarding the 53 animal studies investigating glial cells in migraine, 19 of them explored the role of satellite glial cells and/or Schwann cells in the trigeminal ganglion and/or trigeminal nerve. Additionally, 17 studies highlighted the significance of microglia and/or astrocytes in the trigeminal nucleus caudalis, particularly in relation to central sensitization during migraine chronification. Furthermore, 17 studies examined the involvement of astrocytes and/or microglia in the cortex.

**Conclusion:**

Glial cells, including astrocytes, microglia, satellite glial cells and Schwann cells in the central and peripheral nervous system, participate both in the development as well as chronic progression of migraine in disease-associated regions such as the trigeminovascular system, trigeminal nucleus caudalis and cortex, among other brain regions.

## 1. Introduction

Migraine is a common and debilitating brain disorder that affects about 15% of the population, characterized by moderate to severe headache that typically lasts 4–72 h, often accompanied by other neurological symptoms like nausea, vomiting, photophobia, and phonophobia (International Headache Society, 2018). Depending on the absence or presence of an aura and frequency of attacks, different migraine types such as migraine with aura (MA), migraine without aura (MO), and chronic migraine (CM) were defined (International Headache Society, 2018). Moreover, according to the manifestation of aura, MA was subdivided into migraine with typical or brainstem aura, hemiplegic migraine (HM), and retinal migraine (International Headache Society, 2018). Despite the high prevalence of migraine, its pathological mechanisms are not yet completely understood.

Studies on MA have shown that cortical spreading depression (CSD), a wave of depolarization of neurons and glial cells spreading slowly through the cerebral cortex, followed by a transient silence of the cortical neurons, underlies the aura (Lauritzen, 1994). However, the origin and pathogenesis of headache and other unpleasant neurological symptoms remain to be clarified.

Being first identified in 1979 (Moskowitz et al., 1979), the trigeminovascular system (TVS) is now widely considered to be the essential substrate for migraine headaches. The migraine-related trigeminal sensory pathways consist of the first order neurons located in the trigeminal ganglion (TG) which innervate pain-sensitive intracranial structures such as dura mater and blood vessels, the second-order neurons in the trigeminal nucleus caudalis (TNC) that collect the peripheral trigeminal sensory afferents and project to the brainstem, hypothalamus, basal ganglia, and thalamus, and the third-order neurons in the thalamus that project to related brain regions such as somatosensory cortex, insula and visual cortex (Ashina et al., 2019). In rodents, CSD was shown to initiate activation of trigeminovascular neurons in TG and TNC (Zhang et al., 2011), causing neurogenic inflammation that sensitizes dural nociceptors mediated by vasoactive neuropeptides such as calcitonin gene-related peptide (CGRP), which is now a therapeutic target for migraine (Moskowitz and Macfarlane, 1993; Ashina et al., 2019). The activation of meningeal nociceptors, which is fundamental for the initiation of the headache, in turn sensitizes the second and third neurons at TNC and thalamus, causing allodynia (Ashina et al., 2019).

Many animal migraine models have been developed to help understand the mechanism of migraine. Genetic studies have revealed that familial hemiplegic migraine (FHM) is a monogenic subtype of migraine with aura, and known associated genes include CACNA1A (primarily expressed in neurons), ATP1A2 (primarily expressed in astrocytes), SCN1A (primarily expressed in neurons) and other mutations such as PPRT2 and SLC1A3 (Russell and Ducros, 2011; Nandyala et al., 2023), providing the basis for the current transgenic mouse models of migraine, which have shed some light into the mechanism of migraine. Other commonly used migraine animal models, which mainly rely on inflammatory stimulation of meningeal afferents, nitroglycerin administration or electrical stimulation of trigeminal neurons, focus on the trigeminal sensory processing and have greatly enhanced our understanding of the pathophysiology of migraine-related pain (Harriott et al., 2019a).

Most of the basic research on migraine has mainly centered on neurons. However, given that glial cells have been proven to be fundamental in nociceptive transmission and contribute to the development and maintenance of neuropathic pain (Mika et al., 2013) and chronic pain (Ji et al., 2013), the contribution of glial cells in migraine has gained increasing attention (Kowalska et al., 2021; Amani et al., 2023). In this review, we summarize the studies on the participation of glial cells in the pathophysiology of migraine in human and animal models, in both peripheral and central nervous system (Table 1), in order to better understand the relationship between glial cells and migraine, and prospectively shed light on future therapeutic approaches.

**Table 1:** Summary of studies included in the review.

Grouping of the studies				References
Human studies		Genetic analysis		(Eising et al., 2016; Renthal, 2018)
		Functional imaging		(de la Aleja et al., 2013; Albrecht et al., 2019)
		Serum analysis		(Papandreou et al., 2005)
		Clinical trials		(Kwok et al., 2016)
Animal studies	TG	Satellite glial cells in TG	Glial CGRP receptor expression	(Lennerz et al., 2008; Eftekhari and Edvinsson, 2010; Seiler et al., 2013; Eftekhari et al., 2015; Miller et al., 2016)
			Neuron–Glia crosstalk in TG	(Li et al., 2008; Capuano et al., 2009; Vause and Durham, 2009; Vause and Durham, 2010; De Corato et al., 2011; Neeb et al., 2011; Laursen et al., 2013; Raddant and Russo, 2014)
			Neuron–Glia crosstalk mechanism	(Thalakoti et al., 2007; Damodaram et al., 2009; Suadicani et al., 2010)
		Schwann cells surrounding trigeminal nerve		(De Logu et al., 2022)
		Glia in TG of FHM model		(Ceruti et al., 2011; Hullugundi et al., 2014)
	TNC	NTG model	NTG mouse model	(Long et al., 2018; He et al., 2019; Jing et al., 2019; Long et al., 2020; Jiang et al., 2021; Jing et al., 2021; Wen et al., 2021; Pan et al., 2022)
			NTG rat model	(Chen et al., 2022)
		IS rat model		(Wieseler et al., 2017; Fried et al., 2018; Su et al., 2018; Liu et al., 2018a; Gong et al., 2020; Zhou et al., 2020; Zhu et al., 2021)
	Brain	FHM mouse model	FHM1	(Khennouf et al., 2016; Magni et al., 2019; Dehghani et al., 2021)
			FHM2	(Bøttger et al., 2016; Capuani et al., 2016; Kros et al., 2018; Unekawa et al., 2018; de Iure et al., 2019; Romanos et al., 2020; Smith et al., 2020; Crivellaro et al., 2021; Parker et al., 2021)
			Other gene mutations related to FM	(Suzuki et al., 2010; Brennan et al., 2013)
		Other studies	Hippocampus	(Liu et al., 2018b)
			Cerebellum	(Morara et al., 1998, 2008; Edvinsson et al., 2011)

TG, Trigeminal Ganglia; CGRP, Calcitonin Gene-Related Peptide; FHM, Familial Hemiplegic Migraine; TNC, Trigeminal Nucleus Caudalis; NTG, Nitroglycerin; IS, Inflammatory Soup; CSD, Cortical Spreading Depression; FM, Familial Migraine.

## 2. Search methods

**Figure 1:**
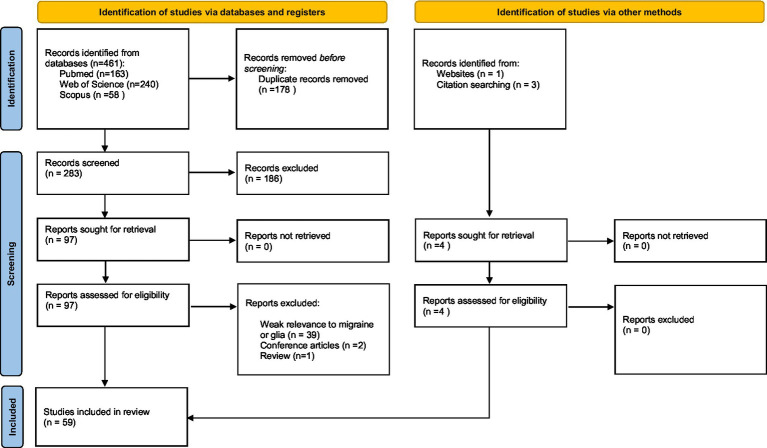
PRISMA flow diagram used for studies selection.

We performed an advanced literature search in PubMed, Scopus and Web of Science for the period January 1, 1990, through November 1, 2022, using search queries including “glia,” “microglia,” “astrocyte,” “oligodendrocyte,” “oligodendrocyte precursor cell,” “satellite glial cell,” “ependymal cell,” “Schwann cell,” and “migraine.” The preliminary evaluation of the eligibility was performed by reviewing the titles and abstracts of the papers found in the literature. The full text of all potentially eligible papers was read and further assessed, and references in eligible papers were also screened. Eligible studies were included according to the following criteria: (1) Human or animal studies were written in English; (2) All patients included in the studies were diagnosed according to “The International Classification of Headache Disorders - ICHD-3″ diagnostic criteria; (3) All animal models used in the studies have been well validated and commonly utilized as preclinical models of migraine. Papers that met the indicated criteria and were found to be relevant to this review were included. The identification, screening and inclusion of articles were performed according to the PRISMA 2020 guideline (Page et al., 2021); the process is shown in the PRISMA chart flow (Figure 1).

## 3. Evidence from animal studies

Animal models of FHM and animal models that mimic the headache attack and chronicity in migraine have been used to study the changes of glial cells associated with pathophysiological mechanisms of migraine. Since the headache produced during acute migraine attack is described to be the most disabling of all the migraine symptoms, this process has become one focus of basic research. Main anatomical areas in the studies include TG and TNC, the most important structures in the pathway of processing facial and cranial pain in migraine, and some investigated the glial cells in cortex, hippocampus and cerebellum.

### 3.1. Studies on glial cells in TG

The trigeminal ganglion (TG) is a cluster of nerve cell bodies that transmit sensory information from the face and brain, forming a fundamental part of the trigeminovascular system. Several studies clearly indicate that TG neurons collaborate closely with non-neuronal satellite glial cells (SGCs), which envelop the neuronal bodies to create a cohesive unit within the ganglion (Hanani, 2005). The crosstalk between neurons and glia involving paracrine signaling and gap junction forms a feedback loop, contributing to the development of neuronal sensitization (Takeda et al., 2009).

#### 3.1.1. Calcitonin gene-related peptide receptor expression in TG

Calcitonin gene-related peptide (CGRP) is mainly synthesized in the TG and released in the TNC (Durham, 2016). It is considered a potential biomarker of migraine headaches since it is shown to be causative in migraine headache with its strong vasodilatory effect (Lassen et al., 2002, 2008). Monoclonal antibodies against CGRP or its receptor such as Galcanezumab (Emgality), Erenumab (Aimovig), and Fremanuzumab (Ajovy) are now used to treat acute migraine headaches (Edvinsson, 2021). CGRP receptor expression is found in the SGCs of the trigeminal ganglion in human (Eftekhari and Edvinsson, 2010), monkey (Eftekhari et al., 2015; Miller et al., 2016), rat (Lennerz et al., 2008; Seiler et al., 2013), and mouse (Ceruti et al., 2011) tissue, pointing to the possibility that CGRP signaling involves both neurons and SGCs within the trigeminal ganglion.

#### 3.1.2. Neuron-glia communication in TG

There is evidence that conditioned medium from activated SGCs augments the evoked release of CGRP by causing excitation of trigeminal neurons (Capuano et al., 2009). Moreover, CGRP released by activated neurons within the trigeminal ganglia was proven to activate SGCs (Li et al., 2008; Vause and Durham, 2009; Vause and Durham, 2010; De Corato et al., 2011), which could contribute to peripheral sensitization in migraine by releasing nitric oxide (NO) (Li et al., 2008). In addition, glial suppressors/modulators and glutamate attenuated the NO release from SGCs harvested from the TG (Laursen et al., 2013). Since NO increases TG neuron excitability, these findings suggest a positive feedback loop and that targeting SGCs may constitute a novel approach to modulate neuronal activity. In addition to this feedback pathway, Raddant and Russo (2014) found that reactive oxygen species released by neurons can activate procalcitonin expression from the CGRP gene in trigeminal glia by a paracrine regulatory mechanism. It is also reported that interleukin 1 beta (IL-1β) activated neurons and glial cells in the cultured rat trigeminal ganglion express more cyclooxygenase 2, which is involved in synthesis of prostaglandin E2. The synthesized prostaglandin E2 in turn increases neuronal release of CGRP (Neeb et al., 2011).

A study by Thalakoti et al. (2007) for the first time reported the neuron–glia crosstalk via gap junctions and paracrine signaling in trigeminal ganglion using an *in vivo* rat model of trigeminal nerve activation. Suadicani et al. (2010) also found out the communication between neurons and SGCs in mouse trigeminal ganglia cultures via bidirectional calcium signaling, mediated by activation of purinergic P2 receptors and gap junctions. A further study showed that an antimigraine drug, Tonabersat, inhibited gap junction communication between neurons and SGCs and prevented the increase of neuron-satellite glia signaling (Damodaram et al., 2009). These findings support a glia-neuron interaction in the trigeminal ganglion during activation of the TVS.

#### 3.1.3. CGRP receptor expression in Schwann cells

In addition to SGCs in the TG, De Logu et al. (2022) found that CGRP released from mouse cutaneous trigeminal fibers targets CGRP receptors on Schwann cells that surround the trigeminal nerve, and evoke periorbital mechanical allodynia.

#### 3.1.4. Studies on glia cells in TG of FHM mouse models

Ceruti et al. (2011) used CaV2.1 α1 R192Q mutant knock-in (KI) mice, which express a human mutation causing familial hemiplegic migraine type 1, to study the crosstalk between neurons and SGCs within the TG. Algogenic mediator bradykinin was found to stimulate neurons in TG to release CGRP, which potentiates P2YR expression on SGCs (Ceruti et al., 2011). In cultures of mouse trigeminal ganglial neurons and SGCs, both basal and bradykinin-stimulated CGRP release were found to be higher in KI mouse cultures, where bradykinin also significantly upregulated the number of SGCs with functional P2Y receptors, indicating that glial P2Y receptors in TG might be a novel player in neuron–glia crosstalk underlying migraine pathophysiology (Ceruti et al., 2011). Using the same mouse model, Hullugundi et al. (2014) patch-clamped trigeminal sensory neurons, and found a lower firing threshold and an increased number of action potentials in KI group compared to wild type group. In addition, in both groups there was a significant firing delay in a minority of neurons, suggesting that these neurons may be indirectly activated via crosstalk between neurons and SGCs (Hullugundi et al., 2014).

### 3.2. Studies on glial cells in trigeminal nucleus caudalis in migraine

Chronic migraine (CM) refers to headache at a frequency of no less than 15 days per month for at least 3 months, of which no less than 8 attacks are migraine or are responsive to migraine-specific treatment (International Headache Society, 2018). CM is a highly disabling migraine subtype and affects up to 2–4% of the global population (Natoli et al., 2010; Stark et al., 2013; Migraine, 2020). Although CM typically progresses from episodic migraine, the exact mechanisms underlying this progression remain to be clarified.

The frequent activation of trigeminal system leads to central sensitization, which is thought to be the underlying mechanism of CM (Dodick and Silberstein, 2006). Central sensitization refers to an increase in the excitability and synaptic efficacy of the central neurons in the trigeminal nociceptive pathway, mainly in the TNC (Mathew, 2011). The most widely used and validated way of modeling the central sensitization in CM includes chronic systemic infusion of nitroglycerin (NTG) and repeated dural application of inflammatory soup (IS) containing histamine, bradykinin, serotonin, prostaglandin E2 (Melo-Carrillo and Lopez-Avila, 2013; Chou and Chen, 2018).

Current studies on the role of glial cells in central sensitization of the TNC mainly focus on microglia, except for one study where the contribution of astrocytes was assessed (Zhou et al., 2020), as discussed later. In most of the studies, the NTG mouse model has mainly been used. A NTG rat model was used in one study and the IS rat model was used in 3 additional studies.

#### 3.2.1. Nitroglycerin-based model

The usage of NTG to induce migraine was inspired by the most frequent side effects of nitrate therapy: headache, which together with accompanying symptoms (nausea, vomiting, phonophobia, photophobia) resembles the features of migraine headache and fulfills the diagnostic criteria of ICHD for migraine in a high percentage of migraineurs (Sances et al., 2004). Besides, NTG infusion also produces effects in blood vessels, nerves and brain areas that are similar to a migraine attack and induces changes in migraine-related biomarkers (Pradhan et al., 2014; Long et al., 2020). Therefore, the NTG model is considered as a reliable model to study migraine-associated headache (Sureda-Gibert et al., 2022).

##### 3.2.1.1. The NTG mouse model

The NTG mouse CM model has mainly been used to illustrate the crosstalk between microglia and neurons in the TNC. Long et al. (2018, 2020) showed that microglia mediate the neuronal excitability marked by elevation of phosphorylated extracellular regulated protein kinases (p-ERK) and CGRP in TNC via the purinergic receptor P2X4 (P2X4R)-p38-mitogen activated protein kinase (p38-MAPK)-brain-derived neurotrophic factor (BDNF) signaling pathway in CM mice. Other microglial receptors and signal pathways that mediate the microglia-neuron crosstalk and lead to central sensitization in CM were also investigated, including: nucleotide oligomerization domain-like receptor protein 3 (NLRP3) inflammasome—IL-1β pathway (He et al., 2019), P2Y12R-RhoA/ROCK pathway (Jing et al., 2019), P2X7R- autophagic flux downregulation-NLRP3 inflammasome pathway (Jiang et al., 2021), microRNA-155-5p - silent information regulator 1 (SIRT1) inhibition (Wen et al., 2021), Sphingosine-1 phosphate receptor 1 (S1PR1)-transcription 3 (STAT3) pathway (Pan et al., 2022). Moreover, activation of glucagon-like peptide-1 receptor (GLP-1R) on glial cells was found to downregulate microglial proinflammatory activation and suppress central sensitization, and the PI3K/Akt pathway was proven to participate in this process (Jing et al., 2021).

##### 3.2.1.2. The NTG rat model

Chen et al. (2022) used the NTG rat model as a migraine model and showed that IL-17 crosses the blood-brain barrier, potentially activating microglia and thus triggered neuroinflammation.

#### 3.2.2. The inflammatory soup rat model

Using IS as a chemical stimulation of the dura was shown to activate TVS. Furthermore, repeated application of IS mimics the chronic central sensitization in CM (Melo-Carrillo and Lopez-Avila, 2013). Studies showed that single or repeated stimulation with IS activates glial cells in TNC and antagonizes toll-like receptor 4 (TLR4)-mediated blocking of IS stimulated facial allodynia (Wieseler et al., 2017; Su et al., 2018). However, Fried reported that glial activation was found only during the chronic stage (after the 10th IS infusion) but not during the episodic stage (after the second IS infusion) (Fried et al., 2018). Zhu et al. (2021) found out increased expression of P2Y14 receptor on microglia in TNC of the IS infused CM rat, which was proven to activate microglia and contribute to central sensitization. Liu et al. (2018a) found increased P2X4-receptor expression in activated TNC microglia in the IS-induced rat CM model, and showed that the P2X4 receptor participates in neuronal excitatory amino acid transporter 3 (EAAT3) regulation via BDNF -tyrosine receptor kinase B (TrkB) signaling.

Zhou et al. (2020) investigated the role of astrocytes in TNC in the central sensitization mechanism of CM in a rat IS model, and found a decrease of astrocytic EAAT2 in CM rats. Moreover, by up-regulating astrocyte EAAT2 they were able to alleviate central sensitization by reducing the synaptic plasticity (Zhou et al., 2020). IL-18-mediated microglia/astrocyte interaction in the medullary dorsal horn was also reported in the IS stimulated migraine rat model (Gong et al., 2020).

### 3.3. Studies on glial cells in the migraine affected brain

The link between CSD and migraine mechanisms has been hypothesized for decades and extensive studies on the essential roles of glia in CSD have been performed. Moreover, it was also shown recently that astrocytes may participate in the CSD triggered TVS activation (Karatas et al., 2013; Zhao and Levy, 2015). However, due to the complexity of the migraine pathophysiology, the CSD animal models are not yet acknowledged as equivalent to migraine models (Harriott et al., 2019b). Therefore, this review excluded studies that investigated the role of glia in CSD but were not conducted using current migraine animal models. The reader is referred to several comprehensive review articles on this aspect (Charles and Baca, 2013; Shibata and Suzuki, 2017; Rovegno and Sáez, 2018).

#### 3.3.1. Studies on glial cells in the brain of FHM mice

##### 3.3.1.1. Studies on glial cells in the brain of FHM1 mice

Magni et al. (2019) described for the first time signs of reactive astrogliosis and microglia activation in the naïve FHM1 mutant mouse brain. In addition, reduced Ca^2+^ responses to somatosensory stimulation in both neurons and astrocytes were reported in FHM1 KI mouse, which may be related to the concomitant impairment of neurovascular coupling (Khennouf et al., 2016). Dehghani et al. (2021) found a higher level of basal neuroinflammation in naïve FHM1 mutant compared to WT mice. Moreover, a single CSD also caused a more profound widespread brain neuroinflammatory response in both hemispheres in KI mice compared to wild type mice. The underlying mechanism was suggested to involve increased neuro-glial communication associated with an enhanced glutamatergic transmission (Dehghani et al., 2021).

##### 3.3.1.2. Studies on glial cells in the brain of FHM2 mice

FHM2 is a subtype of FHM that is caused by mutations of the α2-subunit of the Na,K-ATPase, an isoform that is almost exclusively expressed in astrocytes in adult brain. Patients with FHM2 may exhibit high susceptibility to CSD, resulting in migraine (Capuani et al., 2016), which is also demonstrated in FHM2 mice.

Kros et al. (2018) reported that FHM2-knockin mice (α2^+/G301R^ mice) show an increased susceptibility to both CSD and epileptiform activity, closely replicating symptoms in FHM2 patients. Interestingly, an age-related alteration toward CSD was found in female FHM2 mice, pointing to the influence of female sex hormones on migraine pathophysiology (Kros et al., 2018). Bøttger et al. (2016) reported slower glutamate uptake in hippocampal mixed astrocyte-neuron cultures from this mouse model, where induction of CSD resulted in reduced recovery.

However, the mechanisms by which α2-Na/K ATPase mutations lead to an increased susceptibility to CSD in FHM2 remain incompletely understood. Using heterozygous FHM2-knockin mice (Atp1a2+/R887 mice), researchers found reduced rates of glutamate (Capuani et al., 2016; Parker et al., 2021) and K+ clearance (Capuani et al., 2016) by cortical astrocytes during neuronal activity and reduced density of GLT-1a glutamate transporters in cortical perisynaptic astrocytic processes (Capuani et al., 2016; Parker et al., 2021). A rise in basal glutamate predicted the onset of CSD, suggesting inefficient glutamate clearance as a key mechanism underlying the vulnerability to CSD ignition in migraine. In addition, the enhanced susceptibility to CSD in FHM2 mice was also reported to be due to specific activation of extrasynaptic GluN1-N2B NMDA receptors (Crivellaro et al., 2021). Romanos et al. (2020) used the same model and found that the impaired astrocytic glutamate uptake in the cingulate cortex strongly enhances cortical dendritic excitability, and facilitates migraine-like cranial pain states. The abnormally increased long-term potentiation in the dentate gyrus of FHM 2 mice was suggested to possibly underlie some of the memory deficits observed in FHM (de Iure et al., 2019).

Unekawa et al. (2018) found that the FHM2 simulating Atp1a2-defective mice demonstrated high susceptibility to CSD rather than cortical vasoreactivity, which may differ depending on the knockout strategy for the gene disruption. The study also showed that α2-Na/K ATPase loss altered metabolic gene expression, causing serine and glycine elevation in the brain, and triggered episodic motor paralysis (Smith et al., 2020).

##### 3.3.1.3. Studies on glial cells in the brain of other familial migraine mice models

In addition to FHM1 and 2 mice models, Suzuki et al. (2010) reported the homozygous mutation in SLC4A4, encoding the electrogenic Na + -HCO3− cotransporter NBCe1, to be associated with hemiplegic migraine, and showed that the near total loss of NBCe1B activity in astrocytes is associated with migraine in patients with homozygous NBCe1 mutations. Brennan et al. (2013) reported a family with a mutation in casein kinase Iδ to suffer familial migraine with aura and the corresponding gene mutated mice showed higher sensitivity to pain, lower threshold to CSD and increased spontaneous and evoked calcium signaling in astrocytes.

#### 3.3.2. Other studies on glial cells in migraine brain

##### 3.3.2.1. Hippocampus

Except for the usage of genetic migraine models, one study investigated the changes in hippocampus with rat IS stimulated CM model (Liu et al., 2018b). The study found reduced expression of α7 nicotinic acetylcholine receptor and activation of microglia and astrocytes in CM rat. The activation of α7nAChR decreased the upregulation of astrocytes and microglia through the p-c-Jun N-terminal kinase–mitogen-activated protein kinase signaling pathway and increased the mechanical threshold in the CM rat model (Liu et al., 2018b).

##### 3.3.2.2. Cerebellum

Due to the fundamental role of CGRP in migraine, the distribution and function of CGRP and CGRP receptors within the migraine-related nociceptive areas, such as cerebellum, are also a subject of interest. CGRP containing neurons are widely distributed throughout the central nervous system, with particularly high levels found in the striatum, amygdala, and cerebellum (Edvinsson et al., 2011). Studies have shown that CGRP is exclusively found in the cytoplasm of Purkinje cell bodies (Edvinsson et al., 2011), while CGRP receptors are expressed in Purkinje cells and glial cells (Morara et al., 1998, 2008; Edvinsson et al., 2011). Since the cerebellum is shown to be activated during migraine attacks (Vincent and Hadjikhani, 2007), this suggests the potential involvement of CGRP in mediating the interaction between glial cells and neurons during a migraine attack.

## 4. Evidence from human studies

Several studies have provided evidence for the role of glial cells in migraine. Six human studies on migraineurs based on genetic analyses, functional imaging studies, serum analyses and clinical trials were included here.

### 4.1. Genetic analysis

In general, migraine, except for familial migraine, is a polygenetic disease that involves several cell types. A meta-analysis of genome-wide associated studies from Gormley et al. (2016) indicate that the migraine susceptibility loci is located in vascular and smooth muscle tissues. However, Eising et al. (2016) performed gene set analyses of a migraine genome-wide associated study of 5,954 migraineurs and found that gene sets containing astrocyte- and oligodendrocyte-related genes are associated with both migraine with aura and migraine without aura.

In addition to gross gene expression data, Renthal (2018) analyzed the two published datasets of single cell RNA sequencing from human postmortem brain cortex and determined the enrichment of migraine-associated genes (both familial and common genes) in various brain cell types. They found that the expression of over 40% of known migraine associated genes are cell-type specific, and these migraine susceptibility loci were selectively expressed in neurons, endothelial cells and also glial cells (astrocytes, oligodendrocytes and microglia) (Renthal, 2018).

### 4.2. Functional imaging

de la Aleja et al. (2013) performed proton magnetic resonance spectroscopy on females with migraine with or without aura in the interictal state, quantified glutamate and glutamine in the anterior paracingulate cortex and occipital cortex (OC), and found an increased glutamate/glutamine ratio in the OC of migraine patients. Taking into account the high neuron-to-astrocyte ratio in general in the OC, the high Glu/Gln ratio in migraineurs may suggest a higher neuron/astrocyte ratio and a difference in glutamatergic metabolism between neurons and astrocytes in the OC of migraine patients, which may lead to hyperexcitability and higher susceptibility of the brain to initiate cortical spreading depression in response to migraine triggers (de la Aleja et al., 2013).

In another study, Albrecht et al. (2019) quantified the marker of glial activation in 13 migraineurs with aura in the interictal state with integrated PET/MRI brain scans by measuring the standardized uptake value ratio (SUVR) of its radioligand, [11C]PBR28, and found SUVR elevation in nociceptive processing areas (such as thalamus and primary/secondary somatosensory and insular cortices) and visual cortex, suggesting glia activation and neuroinflammation in migraine with aura. Furthermore, the SUVR elevation in frontoinsular cortex, primary/secondary somatosensory cortices and basal ganglia was positively correlated with the frequency of attacks.

### 4.3. Serum analysis

In a study on serum from children with migraine during or no later than 3 h post pain attack in acute recurrent headache, Papandreou et al. (2005) found a significant elevation of S100β levels. Since S100β is predominately localized in astrocytes and Schwann cells of the nervous system, this study indicates the acute increase of glial activity during migraine.

### 4.4. Clinical trials

One further human study involving the role of glial cells in migraine was a double-blind, randomized, placebo-controlled pilot trial performed by Kwok et al. (2016) to determine the efficacy and safety of ibudilast, a potential glial inhibitor, in chronic migraine. Although no benefit of ibudilast was found, it was spectulated that ibudilast may be insufficient to reverse the long-term changes associated with CM or not have been acting centrally.

## 5. Summary

**Figure 2:**
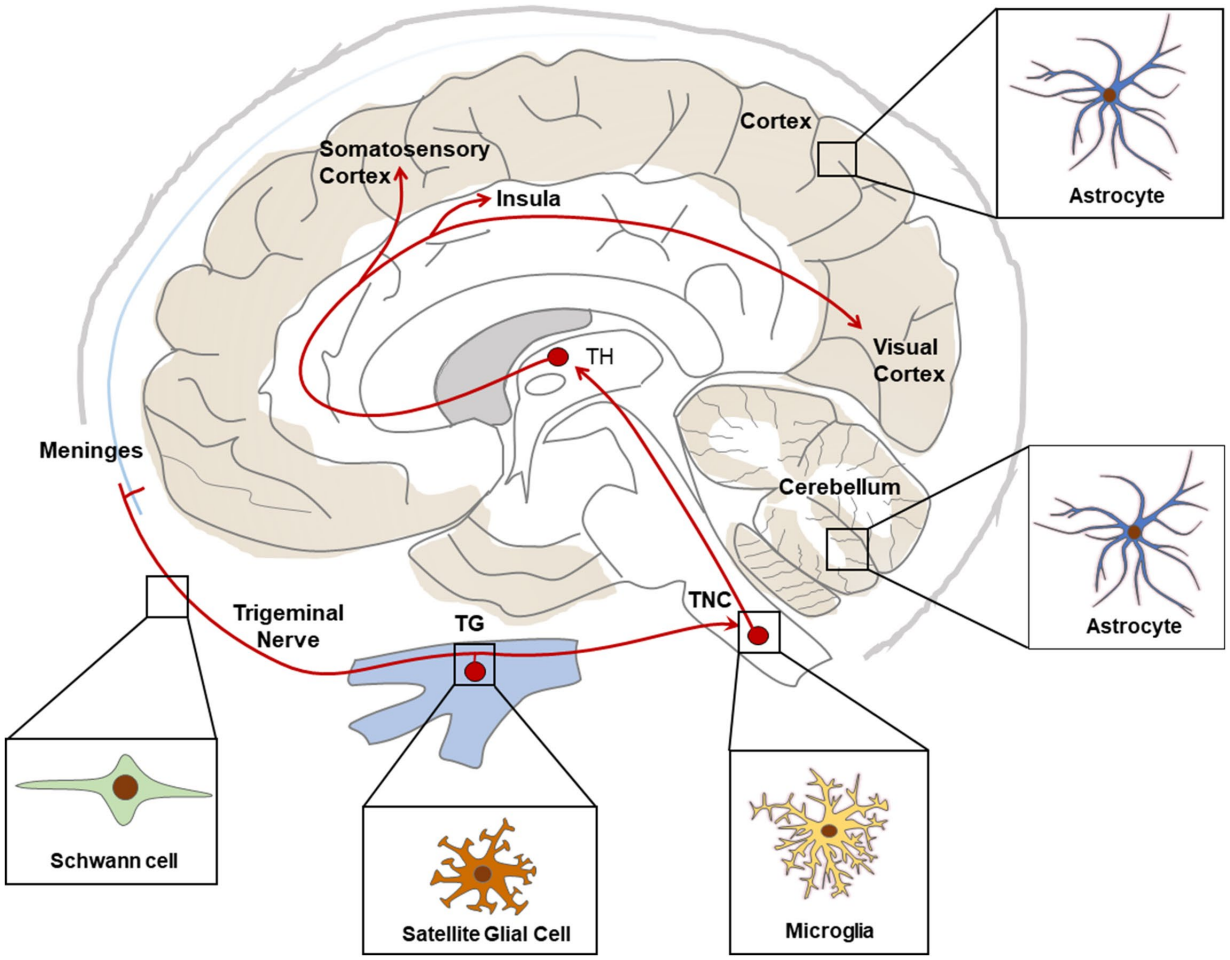
Schematic representation of main glial cell types associated with migraine relevant regions. Red lines show ascending pathways of the trigeminovascular system. TG, Trigeminal Ganglia; TNC, Trigeminal Nucleus Caudalis; TH, Thalamus.

In conclusion, accumulating evidence indicate that glial cells participate actively in the pathophysiology of migraine. In particular, current studies support the role of SGC in neuro-glia interaction and pain signal processing in TG, as well as microglia and astrocytes in the central sensitization of the TNC in CM, and astrocytes in neuronal activity in FHM mouse brains (Figure 2). These findings provide valuable information on pain processing in migraine headache, chronification of migraine and neuro-glia cooperation in FHM, enlightening development of potential therapeutic target of migraine or CM.

Nevertheless, current understanding of the possible pathomechanisms of migraine and the role of glia in this process is still limited. Human studies are restricted due to unpredictable timing of migraine attack and lack of accurate non-invasive approaches with high temporal and spatial resolution. Animal research is also restricted due to the following reasons: (1) The complex features of migraine make it difficult to construct an animal model of migraine that recapitulates all the clinical phenotypes in migraineurs (Chou and Chen, 2018); (2) Though FHM mouse models could theoretically recapitulate the features of FHM, since headache is rather subjective, it is difficult to recognize the migraine attack in animals, which hinders the understanding of the disease. Moreover, FHM is just a rare subtype of migraine with aura that accounts for only a small portion of migraine attacks, and hence it does not reproduce the general features in other migraine subtypes (Dodick, 2018).

Therefore, despite the significant progress that has been made, there is still a substantial requirement to establish new animal models that reproduce more aspects of migraine, and develop more non-invasive studies on migraineurs in order to further elucidate the underlying mechanisms of migraine. Moreover, previous studies have demonstrated the significance of the hypothalamus and dorsal pons in the initiation and sustainment of migraine attacks (Schulte et al., 2020). Hence, investigating the alterations of glial cells in these areas and their potential roles in migraine pathogenesis would be an interesting avenue of research.

## Data availability statement

The original contributions presented in the study are included in the article/Supplementary material, further inquiries can be directed to the corresponding authors.

## Author contributions

SZ conceived the article and screened the papers. SZ and JA wrote the manuscript. CS reviewed and edited the manuscript. All authors contributed to the article and approved the submitted version.

## Funding

We acknowledge support by the German Research Foundation Projekt-Nr. 512648189 and the Open Access Publication Fund of the Thueringer Universitaets- und Landesbibliothek Jena.

## Conflict of interest

The authors declare that the research was conducted in the absence of any commercial or financial relationships that could be construed as a potential conflict of interest.

## Publisher’s note

All claims expressed in this article are solely those of the authors and do not necessarily represent those of their affiliated organizations, or those of the publisher, the editors and the reviewers. Any product that may be evaluated in this article, or claim that may be made by its manufacturer, is not guaranteed or endorsed by the publisher.
